# Glyphosat – Bestimmung von Glyphosat und AMPA in Urin mittels LC-MS/MS: Biomonitoring-Methode

**DOI:** 10.34865/bi107183d10_1or

**Published:** 2025-03-31

**Authors:** Laura Kenny, Craig Sams, Kate Jones, Elisa Polledri, Rosa Mercadante, Silvia Fustinoni, Thomas Göen, Andrea Hartwig, MAK Commission

**Affiliations:** 1 Health and Safety Executive (HSE) Science and Research Centre Harpur Hill SK17 9JN Buxton(Derbyshire); 2 Laboratory of Environmental and Occupational Toxicology. Department of Clinical Sciences and Community Health. University of Milano and Fondazione IRCCS Ca Granda Ospedale Maggiore Policlinico Via Francesco Sforza 35 20122 Milano; 3 Friedrich-Alexander-Universität Erlangen-Nürnberg. Institut und Poliklinik für Arbeits-, Sozial- und Umweltmedizin Henkestraße 9–11 91054 Erlangen Deutschland; 4 Institut für Angewandte Biowissenschaften. Abteilung Lebensmittelchemie und Toxikologie. Karlsruher Institut für Technologie (KIT) Adenauerring 20a, Geb. 50.41 76131 Karlsruhe Deutschland; 5 Ständige Senatskommission zur Prüfung gesundheitsschädlicher Arbeitsstoffe. Deutsche Forschungsgemeinschaft, Kennedyallee 40, 53175 Bonn, Deutschland. Weitere Informationen: Ständige Senatskommission zur Prüfung gesundheitsschädlicher Arbeitsstoffe | DFG

**Keywords:** Glyphosat, AMPA, Biomonitoring, Urin, LC-MS/MS

## Abstract

The working group “Analyses in Biological Materials” of the German Senate Commission for the Investigation of Health Hazards of Chemical Compounds in the Work Area developed and verified the presented biomonitoring method. The aim of this method is the selective and sensitive quantitation of glyphosate (*N*-phosphonomethylglycine) and its only metabolite, aminomethylphosphonic acid (AMPA), in urine. Samples undergo solid-phase extraction prior to liquid chromatography-tandem mass spectrometry using glyphosate-2-^13^C,^15^N and AMPA-^13^C,^15^N,D_2_ as internal standards. Calibration is carried out with urine from persons with no known exposure to glyphosate and AMPA. The procedure has been comprehensively validated and the reliability data have been confirmed by replication and verification of the procedure in a second, independent laboratory. Good precision data with standard deviations of 1.3–9.8% for glyphosate and 1.9–5.4% for AMPA, as well as good accuracy data with mean relative recoveries in the range of 91–102% for glyphosate and 100–106% for AMPA, show that the method provides reliable and accurate analytical results. The method is both selective and sensitive, and the limits of quantitation of 0.1 μg/l for glyphosate and 0.5 μg/l for AMPA are sufficient to determine occupational exposure as well as some of the background exposure in the general population.

## 1 Kenndaten der Methode

**Table:** 

**Matrix**	Urin		
**Analytisches Messprinzip**	Flüssigkeitschromatographie mit Tandem-Massenspektrometrie (LC‑MS/MS)		
**Parameter und entsprechende Arbeitsstoffe**			
**Arbeitsstoff**	**CAS-Nr.**	**Parameter**	**CAS-Nr.**
Glyphosat (*N*‑Phosphonomethylglycin)	1071-83-6	Glyphosat; Aminomethylphosphonsäure (AMPA)	1071-83-6; 1066-51-9
Kaliumglyphosat	70901-12-1; 39600-42-5		
Natriumglyphosat	34494-03-6		
Glyphosatnatriumsalz (2:3)	70393-85-0		
Ammoniumglyphosat	40465-66-5		
Diammoniumglyphosat	69254-40-6		
Triammoniumglyphosat	114370-14-8		
Dimethylammoniumglyphosat	34494-04-7		
Ethanolammoniumglyphosat	40465-76-7		
Isopropylammoniumglyphosat	38641-94-0		
Trimethylsulfoniumglyphosat	81591-81-3		

### Zuverlässigkeitskriterien

#### Glyphosat (Methodenentwicklung auf ZORBAX Eclipse XDB‑C8-Säule)

**Table:** 

Präzision in der Serie:	Standardabweichung (rel.)	*s_w_* = 6,5 % bzw. 2,7 %
	Streubereich	*u* = 15,4 % bzw. 6,11 %
	bei einer dotierten Konzentration von 2 μg oder 7 μg Glyphosat pro Liter Urin und n = 8 oder 10 Bestimmungen	
Präzision von Tag zu Tag:	Standardabweichung (rel.)	*s_w_* = 9,8 %
	Streubereich	*u* = 19,4 %
	bei einer dotierten Konzentration von 4 μg Glyphosat pro Liter Urin und n = 381 Bestimmungen	
Richtigkeit:	Wiederfindung (rel.)	*r* = 94 % bzw. 98 %
	bei einer Sollkonzentration von 4 μg oder 20 μg Glyphosat pro Liter Urin und n = 5 Bestimmungen	
Nachweisgrenze:	0,15 μg Glyphosat pro Liter Urin
Bestimmungsgrenze:	0,5 μg Glyphosat pro Liter Urin

#### Glyphosat (Methodenprüfung auf ZORBAX RR Eclipse XDB‑C8-Säule)

**Table:** 

Präzision in der Serie:	Standardabweichung (rel.)	*s_w_* = 2,4 % bzw. 2,2 %
	Streubereich	*u* = 6,7 % bzw. 6,1 %
	bei einer dotierten Konzentration von 2,5 μg oder 25 μg Glyphosat pro Liter Urin und n = 5 Bestimmungen	
Präzision von Tag zu Tag:	Standardabweichung (rel.)	*s_w_* = 3,4 % bzw. 2,4 %
	Streubereich	*u* = 14,6 % bzw. 10,3 %
	bei einer dotierten Konzentration von 2,5 μg oder 25 μg Glyphosat pro Liter Urin und n = 3 Bestimmungen	
Richtigkeit:	Wiederfindung (rel.)	*r* = 99 % bzw. 100 %
	bei einer Sollkonzentration von 2,5 μg oder 25 μg Glyphosat pro Liter Urin und n = 3 Bestimmungen	
Nachweisgrenze:	0,1 μg Glyphosat pro Liter Urin
Bestimmungsgrenze:	0,5 μg Glyphosat pro Liter Urin

#### AMPA (Methodenprüfung auf ZORBAX RR Eclipse XDB‑C8-Säule)

**Table:** 

Präzision in der Serie:	Standardabweichung (rel.)	*s_w_* = 3,5 % bzw. 1,9 %
	Streubereich	*u* = 9,7 % bzw. 5,3 %
	bei einer dotierten Konzentration von 2,5 μg oder 25 μg AMPA pro Liter Urin und n = 5 Bestimmungen	
Präzision von Tag zu Tag:	Standardabweichung (rel.)	*s_w_* = 5,4 % bzw. 2,6 %
	Streubereich	*u* = 23,2 % bzw. 11,2 %
	bei einer dotierten Konzentration von 2,5 μg oder 25 μg AMPA pro Liter Urin und n = 3 Bestimmungen	
Richtigkeit:	Wiederfindung (rel.)	*r* = 102 % bzw. 102 %
	bei einer Sollkonzentration von 2,5 μg oder 25 μg AMPA pro Liter Urin und n = 3 Bestimmungen
Nachweisgrenze:	0,1 μg AMPA pro Liter Urin
Bestimmungsgrenze:	0,5 μg AMPA pro Liter Urin

#### Glyphosat (Methodenprüfung auf Raptor Polar X-Säule)

**Table:** 

Präzision in der Serie:	Standardabweichung (rel.)	*s_w_* = 2,1 % bzw. 1,3 %
	Streubereich	*u* = 5,8 % bzw. 3,6 %
	bei einer dotierten Konzentration von 2,5 μg oder 25 μg Glyphosat pro Liter Urin und n = 5 Bestimmungen	
Präzision von Tag zu Tag:	Standardabweichung (rel.)	*s_w_* = 4,3 % bzw. 2,1 %
	Streubereich	*u* = 18,5 % bzw. 9,0 %
	bei einer dotierten Konzentration von 2,5 μg oder 25 μg Glyphosat pro Liter Urin und n = 3 Bestimmungen	
Richtigkeit:	Wiederfindung (rel.)	*r* = 102 % bzw. 100 %
	bei einer Sollkonzentration von 2,5 μg oder 25 μg Glyphosat pro Liter Urin und n = 3 Bestimmungen	
Nachweisgrenze:	0,05 μg Glyphosat pro Liter Urin
Bestimmungsgrenze:	0,1 μg Glyphosat pro Liter Urin

#### AMPA (Methodenprüfung auf Raptor Polar X-Säule)

**Table:** 

Präzision in der Serie:	Standardabweichung (rel.)	*s_w_* = 2,8 % bzw. 3,8 %
	Streubereich	*u* = 7,8 % bzw. 10,5 %
	bei einer dotierten Konzentration von 2,5 μg oder 25 μg AMPA pro Liter Urin und n = 5 Bestimmungen	
Präzision von Tag zu Tag:	Standardabweichung (rel.)	*s_w_* = 3,1 % bzw. 4,2 %
	Streubereich	*u* = 13,3 % bzw. 18,1 %
	bei einer dotierten Konzentration von 2,5 μg oder 25 μg AMPA pro Liter Urin und n = 3 Bestimmungen	
Richtigkeit:	Wiederfindung (rel.)	*r* = 106 % bzw. 100 %
	bei einer Sollkonzentration von 2,5 μg oder 25 μg AMPA pro Liter Urin und n = 3 Bestimmungen	
Nachweisgrenze:	0,1 μg AMPA pro Liter Urin
Bestimmungsgrenze:	0,5 μg AMPA pro Liter Urin

## 2 Allgemeine Informationen zu Glyphosat und AMPA

Die Substanz *N*‑Phosphonomethylglycin, besser bekannt unter dem Namen Glyphosat, wurde im Jahr 1950 von der Schweizer Pharmafirma Cilag entwickelt und später zunächst als Wasserenthärtungsmittel auf Phosphonsäurebasis eingesetzt (Székács und Darvas 2012). Das US-amerikanische Unternehmen Monsanto erkannte das Potenzial des Glyphosats für die Unkrautbekämpfung und ließ sich die Substanz im Jahr 1974 als Breitbandherbizid patentieren (Dill et al. 2010).

**Abb. 1:**
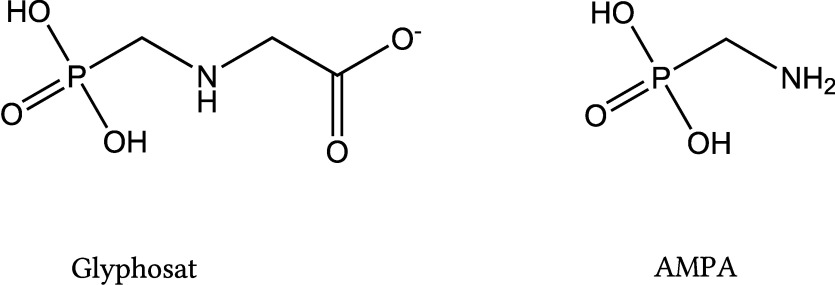
Strukturformeln von Glyphosat und AMPA

Reines Glyphosat in protonierter Form ist ein weißer, geruchloser Feststoff (Strukturformel siehe Abbildung 1), der eine geringe Wasserlöslichkeit aufweist (11,6 g/l bei 25 °C) (IARC 2017). Aus diesem Grund werden in Herbiziden zumeist Salze des Glyphosats verwendet und verschiedene Adjuvantien zur Steigerung der Wirksamkeit zugesetzt. In Tabelle 1 sind die wichtigsten Glyphosatsalze, die als Wirkstoffe in Glyphosatformulierungen eingesetzt werden, aufgeführt. Als Adjuvantien kommen vor allem Tenside wie polyethoxyliertes Talgamin (Martins-Gomes et al. 2022), aber auch Antischaummittel, Abdriftkontrollmittel oder Wasser-Konditionierungsmittel zur Anwendung (Dill et al. 2010).

**Tab. 1:** Glyphosatsalze, die als Wirkstoffe in Glyphosatformulierungen eingesetzt werden (nach ATSDR 2020)

**Substanz**	**CAS‑Nr.**	**Kation**
Kaliumglyphosat	70901-12-1; 39600-42-5	K^+^
Natriumglyphosat	34494-03-6	Na^+^
Glyphosatnatriumsalz (2:3)	70393-85-0	Na^+^
Ammoniumglyphosat	40465-66-5	NH_4_^+^
Diammoniumglyphosat	69254-40-6	NH_4_^+^
Triammoniumglyphosat	114370-14-8	NH_4_^+^
Dimethylammoniumglyphosat	34494-04-7	NH_2_(CH_3_)_2_^+^
Ethanolammoniumglyphosat	40465-76-7	NH_3_(CH_2_CH_2_OH)^+^
Isopropylammoniumglyphosat	38641-94-0	NH_3_CH(CH_3_)_2_^+^
Trimethylsulfoniumglyphosat	81591-81-3	S(CH_3_)_3_^+^

Neben Roundup^®^ sind derzeit mehr als 750 glyphosathaltige Produkte auf dem Markt, die mit einer Absatzmenge von ca. 800 000 t pro Jahr die weltweit am häufigsten verwendeten Herbizide darstellen (Antier et al. 2020; Polledri et al. 2023). Diese Herbizide auf Glyphosatbasis werden zu 90 % in der Landwirtschaft eingesetzt, aber auch im Garten- und Weinbau, in der Forstwirtschaft, an Bahntrassen und Straßenrändern sowie auf öffentlichen Grünflächen und in privaten Gärten (Dill et al. 2010; Duke und Powles 2008; Jaworski 1972).

Die herbizide Wirkung von Glyphosat beruht auf der Hemmung der 5‑Enolpyruvylshikimat-3‑phosphat-Synthase (EPSPS), einem Enzym, das nur in Pflanzen, Pilzen und einigen Mikroorganismen vorkommt. In Pflanzen ist EPSPS für die Biosynthese aromatischer Aminosäuren essenziell, so dass die Aufnahme von Glyphosat zu einer Hemmung der Proteinbiosynthese und schließlich zum Absterben der Pflanze führt (Dill et al. 2010; Jaworski 1972).

Versuche an Ratten haben gezeigt, dass bei oral verabreichten Dosen von 1–1000 mg Glyphosat/kg Körpergewicht etwa 20 % der oral verabreichten Menge resorbiert werden (EFSA 2023; EFSA et al. 2023). Das aufgenommene Glyphosat verteilt sich im gesamten Körper, wobei sich die höchsten Konzentrationen in den Knochen, den Nieren und der Leber finden. Bei Säugetieren gibt es jedoch keine Hinweise auf eine Bioakkumulation. Innerhalb von 48 Stunden wird das Glyphosat größtenteils ausgeschieden: das nicht resorbierte Glyphosat mit den Faeces, das resorbierte Glyphosat überwiegend unmetabolisiert mit dem Urin (EFSA et al. 2023). Bisherige Studien deuten darauf hin, dass Glyphosat im Organismus von Säugetieren nur in sehr geringem Umfang metabolisiert wird und AMPA (Strukturformel siehe Abbildung 1) den einzigen Metaboliten darstellt. Zoller et al. (2020) wiesen an zwölf Probanden nach, dass 48 Stunden nach Verzehr Glyphosat- und AMPA-belasteter Lebensmittel nur 1 % der Glyphosatdosis mit dem Urin ausgeschieden wurde und die ausgeschiedenen AMPA-Konzentrationen etwa 0,2 % der aufgenommenen Glyphosatmenge entsprachen. Nach derzeitigem Kenntnisstand wird Glyphosat biphasisch eliminiert, mit Halbwertszeiten von 2,1–7,5 h sowie von 69–337 h (EFSA 2015 a). Bei Annahme einer Kinetik erster Ordnung werden für den Menschen Eliminationshalbwertzeiten von 5,5 h (Connolly et al. 2019), 4–17 h (Faniband et al. 2017) sowie 9 h (Zoller et al. 2020) berichtet.

Das Hauptabbauprodukt des Glyphosats in der Umwelt ist AMPA. So können Glyphosat und AMPA sowohl in Nutzpflanzen, die mit glyphosathaltigen Herbiziden behandelt wurden, als auch in Oberflächengewässern, Grundwasser und Böden nachgewiesen werden (Borggaard und Gimsing 2008). Demzufolge kann die Hintergrundbelastung der Allgemeinbevölkerung gegen Glyphosat und AMPA sowohl aus der Nahrung als auch aus der Umwelt stammen. Generell wird angenommen, dass nur ein geringer Anteil des im Urin der Allgemeinbevölkerung nachgewiesenen AMPAs aus der metabolischen Umsetzung des Glyphosats stammt und der überwiegende Anteil im Rahmen der Ernährung aufgenommen wird (EFSA 2015 a, b). Zudem kann AMPA auch durch mikrobiellen und photochemischen Abbau von Aminophosphonsäuren, die Bestandteil von Tensiden, Flammschutzmitteln und anderen Industriechemikalien sein können, gebildet werden (Grandcoin et al. 2017; Struger et al. 2015). Diese Prozesse können in Verbindung mit dem eher langsamen Abbau von AMPA in der Umwelt zu dessen Anreicherung in pflanzlichen und tierischen Produkten führen (van Bruggen et al. 2018).

Die Hintergrundbelastung der Allgemeinbevölkerung mit Glyphosat und AMPA wurde bereits in mehreren Biomonitoringstudien untersucht. Es zeigte sich eine generelle Belastung, wobei zumeist eher niedrige Gehalte im Bereich der Bestimmungsgrenze gefunden wurden. Eine Übersicht über Glyphosat- und AMPA-Konzentrationen in Urinproben der deutschen Allgemeinbevölkerung ist in der 2023 veröffentlichten Methode der Kommission zur Bestimmung von Glyphosat und AMPA in Urin mittels GC‑MS/MS (Hoppe et al. 2023) enthalten. In einer Übersichtsarbeit von Gillezeau et al. (2019) wurden Daten zur Glyphosat- und AMPA-Belastung der beruflich nicht belasteten Allgemeinbevölkerung und exponierter Arbeiter aus unterschiedlichen Ländern zusammengestellt. Dabei zeigten sich bei den nicht beruflich exponierten Personen sehr große regionale Unterschiede mit mittleren Glyphosatgehalten zwischen 0,16 μg und 4 μg pro Liter Urin. In Gegenden, in denen glyphosathaltige Herbizide mit dem Flugzeug versprüht wurden, lag die Glyphosatkonzentration im Urin im Mittel bei 7,6 μg/l (Gillezeau et al. 2019; Varona et al. 2009).

Bei Arbeitern erfolgt die Exposition gegen Glyphosat hauptsächlich inhalativ und dermal. Gillezeau et al. (2019) stellten Studienergebnisse aus Land- und Forstwirtschaft sowie der Landschaftsgärtnerei zusammen und berichten mittlere Glyphosatkonzentrationen im Urin von 0,26 bis 73,5 μg/l, wobei die maximale Belastung von 233 μg/l bei einem Landwirt in den USA gefunden wurde (Acquavella et al. 2004). Bei Beschäftigten einer Glyphosat-Fabrik in China lagen die gemessenen Glyphosatgehalte bei < 20–17 200 μg/l Urin und die AMPA-Gehalte bei < 10–2730 μg/l Urin (Zhang et al. 2020). Glyphosat- und AMPA-Konzentrationen im Urin von Beschäftigten verschiedener Berufe zusammen mit der Nachweishäufigkeit und der verwendeten Analysenmethode sind auch in Hoppe et al. (2023) zu finden.

In der Europäischen Union (EU) gab es bislang drei Risikobewertungen von Glyphosat, die 2002 zunächst zur Erst­zulassung von Glyphosat in der EU führten, 2017 kam es zur Erneuerung der Genehmigung und 2023 führte die neueste Bewertung der Europäischen Behörde für Lebensmittelsicherheit (*European Food Safety Authority, *EFSA) und der Europäischen Chemikalienagentur (*European Chemicals Agency*, ECHA) zu einer Verlängerung der Glyphosat­zulassung bis Ende 2033 (European Commission 2023). Die Internationale Agentur für Krebsforschung (*International Agency for Research on Cancer*, IARC) hat Glyphosat als wahrscheinlich kanzerogen für den Menschen (Gruppe 2A) eingestuft (IARC 2017). Im Gegensatz dazu kam die ECHA bei ihrer Risikobewertung im Jahr 2022 zu dem Schluss, dass Glyphosat die wissenschaftlichen Kriterien für eine Einstufung als krebserzeugender, erbgutverändernder oder fortpflanzungsgefährdender Stoff nicht erfüllt (RAC 2022). Die Diskussion über eine mögliche Kanzerogenität und über andere gesundheitliche Risiken (Teratogenität und hormonelle Störungen) ist noch nicht abgeschlossen (van Bruggen et al. 2018; Galli et al. 2024; US EPA 2017). Von der Kommission wurde Glyphosat noch nicht bewertet.

## 3 Grundlage des Verfahrens

Die hier beschriebene Analysenmethode ermöglicht die Quantifizierung des Herbizids Glyphosat und seines einzigen Metaboliten AMPA in Urin. Nach Zugabe von markierten internen Standards (Glyphosat‑2‑^13^C,^15^N; AMPA‑^13^C,^15^N,D_2_), werden die Proben mittels Festphasenextraktion aufgearbeitet. Die Analytkonzentrationen werden mittels Flüssigkeits­chromatographie mit tandem-massenspektrometrischer Detektion bestimmt. Kalibrierstandards werden im Urin von Personen ohne bekannte Glyphosat- oder AMPA-Exposition angesetzt und wie die zu analysierenden Proben aufgearbeitet.

## 4 Geräte, Chemikalien und Lösungen

### 4.1 Geräte

#### Methodenentwicklung

- Flüssigkeitschromatograph (z. B. Shimadzu LC‑20AB, Shimadzu UK Limited, Milton Keynes, Vereinigtes Königreich) mit Tandem-Massenspektrometer (z. B. AB SCIEX API 3200, AB SCIEX LLC, Framingham, MA, USA)
- Analytische Säule (z. B. ZORBAX Eclipse XDB‑C8, 4,6 × 150 mm, 5 μm, Nr. 993967-906, Agilent Technologies LDA UK Limited, Stockport, Vereinigtes Königreich)
- C18-Vorsäule (z. B. SecurityGuard Cartridges, AQ C18 4 × 2.0 mm, Nr. AJ0‑7510‑S, Phenomenex Ltd, Macclesfield, Vereinigtes Königreich)
- System zur Automatisierung der Festphasenextraktion (z. B. ASPEC^®^ GX‑271, Nr. 2614101, Gilson UK, Dunstable, Vereinigtes Königreich)
- SPE-Kartuschen (z. B. Strata SAX, 55 μm, 70 Å, 100 mg/1 ml, Nr. 8B‑S008‑EAK, Phenomenex Ltd, Macclesfield, Vereinigtes Königreich)

#### Methodenprüfung

- Flüssigkeitschromatograph (z. B. Agilent 1260 Infinity III LC System, Agilent Technologies Italia S.p.A., Cernusco sul Naviglio, Italien) mit Tandem-Massenspektrometer (z. B. QTRAP^®^ 5500 LC‑MS/MS System, AB SCIEX LLC, Framingham, MA, USA)
- Analytische Säule I (z. B. ZORBAX RR Eclipse XDB‑C8, 2,1 × 150 mm, 3,5 μm, Nr. 930990‑906, Agilent Technologies Italia S.p.A., Cernusco sul Naviglio, Italien)
- Analytische Säule II (z. B. Raptor Polar X, 50 × 2,1 mm, 2,7 μm, Nr. 9311A52, Restek S.r.l., Cernusco sul Naviglio, Italien)
- SPE-Kartuschen (z. B. SampliQ Silica SAX, 500 mg/3 ml, Nr. 5982‑2035, Agilent Technologies Italia S.p.A., Cernusco sul Naviglio, Italien)

#### Allgemeine Ausrüstung

- Laborzentrifuge (z. B. Heraeus Deutschland GmbH & Co. KG, Hanau)
- Wasseraufbereitungssystem (z. B. Milli‑Q^®^, Merck KGaA, Darmstadt)
- Heizblock mit Stickstoff-Evaporator (z. B. Pierce Reacti‑Therm III 18935 Heiz/Rühr-Modul mit Reacti‑Vap III 18785 Aufsatz, Pierce, Rockford, IL, USA)
- Analysenwaage (z. B. Denver Instrument A‑200DS, Nr. 22827, American Laboratory Trading, East Lyme, CT, USA)
- 5‑ml-, 25‑ml-, 50‑ml-, 150‑ml-, 500‑ml- und 1000‑ml-Messkolben (z. B. BRAND GMBH + CO KG, Wertheim)
- Variabel einstellbare Pipetten, 10–100 μl und 100–1000 μl (z. B. Gilson UK, Dunstable, Vereinigtes Königreich)
- Multipette^®^ mit 20‑μl-, 100‑μl- und 200‑μl-Combitips^®^ (z. B. Eppendorf AG, Hamburg)
- 3,5-ml-Einwegpipetten (z. B. Sarstedt AG & Co. KG, Nümbrecht)
- Messzylinder (z. B. BRAND GMBH + CO KG, Wertheim)
- Kunststoffvials mit vorgeformten Polypropylen-Inserts und Bördelkappen (z. B. Nr. 24651, Restek GmbH, Bad Homburg vor der Höhe)
- 5‑ml-Polypropylenröhrchen, 10 × 75 mm (z. B. Nr. 1E8Y.1, Carl Roth GmbH + Co. KG, Karlsruhe)
- 5‑ml-Polypropylenröhrchen, 12 × 55 mm (z. B. Nr. 115201, Greiner Bio-One International GmbH, Kremsmünster, Österreich)
- Urinbecher mit Schraubverschluss (z. B. Sarstedt AG & Co. KG, Nümbrecht)

### 4.2 Chemikalien

Wenn nicht anders angegeben, sind alle genannten Chemikalien mindestens in p. a.-Qualität zu verwenden.

#### Methodenentwicklung

- Glyphosat PESTANAL^®^, ≥ 99 % (z. B. Nr. 45521, Merck KGaA, Darmstadt)
- Glyphosat-2‑^13^C,^15^N (z. B. Nr. 90479, Merck KGaA, Darmstadt)
- Acetonitril für die HPLC (z. B. Nr. RH1015, Rathburn Chemicals Ltd., Walkerburn, Vereinigtes Königreich)
- Ameisensäure (z. B. Nr. 5.43804, Merck KGaA, Darmstadt)
- Methanol für die HPLC (z. B. Nr. RH1019, Rathburn Chemicals Ltd., Walkerburn, Vereinigtes Königreich)
- Hochreines Wasser (z. B. Milli‑Q^®^, Merck KGaA, Darmstadt)
- Stickstoff 5.0 (Air Liquide Deutschland GmbH, Düsseldorf)
- Urin von Personen ohne bekannte Exposition gegen Glyphosat

#### Methodenprüfung

- Aminomethylphosphonsäure (AMPA) ≥ 99 % (z. B. Nr. 324817, Merck KGaA, Darmstadt)
- Aminomethylphosphonsäure‑^13^C,^15^N,D_2_, 100 μg/ml in Wasser (z. B. Nr. CDNLM‑6786‑10, Cerilliant^®^, Merck KGaA, Darmstadt)
- Glyphosat PESTANAL^®^, ≥ 99 % (z. B. Nr. 45521, Merck KGaA, Darmstadt)
- Glyphosat-2‑^13^C,^15^N (z. B. Nr. 90479, Merck KGaA, Darmstadt)
- Acetonitril für die HPLC (z. B. Nr. 34851, Merck KGaA, Darmstadt)
- Ameisensäure (z. B. Nr. 1.11670, Merck KGaA, Darmstadt)
- Methanol für die HPLC (z. B. Nr. 34860‑R, Merck KGaA, Darmstadt)
- Hochreines Wasser (z. B. Milli‑Q^®^, Merck KGaA, Darmstadt)
- Stickstoff 5.0 (Air Liquide Deutschland GmbH, Düsseldorf)
- Urin von Personen ohne bekannte Exposition gegen Glyphosat oder AMPA

### 4.3 Lösungen

#### Methodenentwicklung (ZORBAX Eclipse XDB‑C8-Säule) und Methodenprüfung (ZORBAX RR Eclipse XDB‑C8-Säule)

- 10%ige Ameisensäure in Methanol
  In einem 150‑ml‑Messkolben wird Methanol vorgelegt und 15 ml Ameisensäure werden zugegeben. Der Kolben wird anschließend bis zur Markierung mit Methanol aufgefüllt und die Lösung gut gemischt.

Die Lösung muss arbeitstäglich frisch hergestellt werden.

- Eluent A (0,1%ige Ameisensäure in Wasser)
  In einem 1000‑ml‑Messkolben wird hochreines Wasser vorgelegt und 1 ml Ameisensäure zugegeben. Anschließend wird der Kolben bis zur Markierung mit hochreinem Wasser aufgefüllt und die Lösung gut gemischt.

Die Lösung ist mindestens eine Woche bei Raumtemperatur stabil.

#### Methodenprüfung (Raptor Polar X-Säule)

- 0,1%ige Ameisensäure in Wasser
  In einem 1000‑ml‑Messkolben wird hochreines Wasser vorgelegt und 1 ml Ameisensäure zugegeben. Anschließend wird der Kolben bis zur Markierung mit hochreinem Wasser aufgefüllt und die Lösung gut gemischt.

Die Lösung ist mindestens eine Woche bei Raumtemperatur stabil.

- Eluent A (0,5%ige Ameisensäure in Wasser)
  In einem 1000‑ml‑Messkolben wird hochreines Wasser vorgelegt und es werden 5 ml Ameisensäure zugegeben. Der Messkolben wird anschließend bis zur Markierung mit hochreinem Wasser aufgefüllt und die Lösung gut gemischt.

Die Lösung ist mindestens eine Woche bei Raumtemperatur stabil.

- Eluent B (0,5%ige Ameisensäure in Acetonitril)
  In einem 1000‑ml‑Messkolben wird Acetonitril vorgelegt und es werden 5 ml Ameisensäure zugegeben. Anschließend wird der Messkolben bis zur Markierung mit Acetonitril aufgefüllt und die Lösung gut gemischt.

Die Lösung ist mindestens eine Woche bei Raumtemperatur stabil.

### 4.4 Interne Standards (ISTDs)

#### Methodenentwicklung

- Glyphosat-2‑^13^C,^15^N‑Stammlösung (200 mg/l)
  In einen 25‑ml‑Messkolben werden etwa 5 mg Glyphosat-2‑^13^C,^15^N exakt eingewogen und in hochreinem Wasser gelöst. Anschließend wird der Messkolben mit hochreinem Wasser bis zur Markierung aufgefüllt.
- Glyphosat-2‑^13^C,^15^N‑Dotierlösung (0,12 mg/l)
  In einen 25‑ml-Messkolben, in dem etwas hochreines Wasser vorgelegt wurde, werden 15 μl der Glyphosat‑2‑^13^C,^15^N‑Stammlösung pipettiert. Der Messkolben wird dann mit hochreinem Wasser bis zur Markierung aufgefüllt.

Die Stammlösung und die Dotierlösung werden sofort in Kunststoffröhrchen umgefüllt und bei –20 °C gelagert. Unter diesen Lagerungsbedingungen ist der Analyt mindestens ein Jahr stabil (Hoppe et al. 2023).

#### Methodenprüfung

- Glyphosat-2‑^13^C,^15^N‑Stammlösung (100 mg/l)
  1 mg Glyphosat-2‑^13^C,^15^N werden in einen 10‑ml-Messkolben eingewogen und in hochreinem Wasser gelöst. Anschließend wird der Messkolben bis zur Markierung mit hochreinem Wasser aufgefüllt.
- Glyphosat-2‑^13^C,^15^N- und AMPA‑^13^C,^15^N,D_2_‑Dotierlösung (je 2,5 mg/l)
  In einen 5‑ml‑Messkolben werden 125 μl der Glyphosat-2‑^13^C,^15^N‑Stammlösung und 125 μl der AMPA‑^13^C,^15^N,D_2_-Lösung (100 mg/l) pipettiert. Der Kolben wird anschließend mit hochreinem Wasser bis zur Markierung aufgefüllt.

Die Stammlösung und die Dotierlösung werden sofort in Kunststoffröhrchen umgefüllt und bei −20 °C gelagert. Unter diesen Lagerungsbedingungen sind die Analyten mindestens ein Jahr stabil (Hoppe et al. 2023).

### 4.5 Kalibrierstandards

Für die Herstellung der Kalibrierstandards wird der Urin von Personen verwendet, die nicht gegen Glyphosat oder AMPA exponiert sind. Die Kalibrierstandards werden für jede Analysenserie neu hergestellt und in der gleichen Weise wie die zu analysierenden Proben gemäß Abschnitt 5.2 aufgearbeitet.

#### Methodenentwicklung

- Glyphosat-Stammlösung (700 mg/l)
  17,5 mg Glyphosat werden in einen 25‑ml-Messkolben eingewogen und in hochreinem Wasser gelöst. Der Messkolben wird anschließend mit hochreinem Wasser bis zur Markierung aufgefüllt.
- Glyphosat-Arbeitslösung (0,4 mg/l)
  28,6 μl der Glyphosat-Stammlösung werden in einen 50‑ml-Messkolben pipettiert. Der Messkolben wird anschließend mit hochreinem Wasser bis zur Markierung aufgefüllt.

Die Stamm- und die Arbeitslösung werden sofort in Kunststoffröhrchen umgefüllt und bei –20 °C gelagert. Unter diesen Lagerungsbedingungen ist der Analyt mindestens ein Jahr stabil (Hoppe et al. 2023).

- Glyphosat-Dotierlösung (20 μg/l)
  In einem Kunststoffröhrchen werden 950 μl Urin einer Person ohne bekannte Glyphosatexposition vorgelegt und 50 μl der Glyphosat-Arbeitslösung zupipettiert.

Die Dotierlösung wird arbeitstäglich frisch angesetzt.

Kalibrierstandards in einem Konzentrationsbereich bis 20 μg/l werden in 5‑ml-Polypropylenröhrchen gemäß dem in Tabelle 2 dargestellten Pipettierschema hergestellt.

**Tab. 2:** Pipettierschema für die Herstellung der Kalibrierstandards für die Bestimmung von Glyphosat in Urin (Methodenentwicklung)

Kalibrierstandard	Dotierlösung [μl]	Urin [μl]	Glyphosatkonzentration [μg/l]
0	–	200	0
1	20	180	2
2	40	160	4
3	80	120	8
4	120	80	12
5	160	40	16
6	200	0	20

#### Methodenprüfung

- Glyphosat-Stammlösung (1000 mg/l)
  5 mg Glyphosat werden in einen 5‑ml‑Messkolben eingewogen und in hochreinem Wasser gelöst. Anschließend wird der Messkolben mit hochreinem Wasser bis zur Markierung aufgefüllt.
- AMPA-Stammlösung (1000 mg/l)
  5 mg AMPA werden in einen 5‑ml‑Messkolben eingewogen und in hochreinem Wasser gelöst. Anschließend wird der Messkolben mit hochreinem Wasser bis zur Markierung aufgefüllt.
- Arbeitslösung (100 mg/l)
  In einem Polypropylenröhrchen werden 400 μl der Glyphosat-Stammlösung und 400 μl der AMPA-Stammlösung zu 3200 μl hochreinem Wasser pipettiert.
- Dotierlösung I (250 μg/l)
  In einem Polypropylenröhrchen werden 10 μl der Arbeitslösung zu 3390 μl hochreinem Wasser pipettiert.
- Dotierlösung II (25 μg/l)
  In einen 5‑ml‑Messkolben werden 500 μl der Dotierlösung I pipettiert. Der Kolben wird anschließend mit hochreinem Wasser bis zur Markierung aufgefüllt.
- Dotierlösung III (2,5 μg/l)
  In einen 5‑ml‑Messkolben werden 500 μl der Dotierlösung II pipettiert. Anschließend wird der Kolben bis zur Markierung mit hochreinem Wasser aufgefüllt.

Zur langfristigen Lagerung werden die Stamm-, Arbeits- und Dotierlösungen von Glyphosat und AMPA sofort in Kunststoffröhrchen umgefüllt und bei −20 °C tiefgefroren. Unter diesen Lagerungsbedingungen sind die Analyten mindestens sechs Monate stabil.

Kalibrierstandards in Urin werden in 5‑ml‑Polypropylenröhrchen nach dem in Tabelle 3 angegebenen Pipettierschema hergestellt.

**Tab. 3:** Pipettierschema für die Herstellung von Kalibrierstandards für die Bestimmung von Glyphosat und AMPA in Urin (Methodenprüfung)

Kalibrierstandard	Dotierlösung I [μl]	Dotierlösung II [μl]	Dotierlösung III [μl]	Urin [μl]	Glyphosat/AMPA-Konzentration [μg/l]
0	–	–	–	1000	0
1	–	–	40	960	0,1
2	–	20	–	980	0,5
3	–	40	–	960	1
4	–	80	–	920	2
5	20	–	–	980	5
6	40	–	–	960	10
7	80	–	–	920	20
8	160	–	–	840	40

## 5 Probenahme und Probenaufbereitung

### 5.1 Probenahme

Die Urinproben werden in geeigneten Plastikurinbechern gesammelt und bis zur Aufarbeitung bei −20 °C gelagert.

### 5.2 Probenaufbereitung

#### Methodenentwicklung

Vor der Analyse werden die Urinproben bei Raumtemperatur aufgetaut und gut durchgemischt. Alle Proben werden doppelt bestimmt. 200 μl Urin werden in ein Polypropylenröhrchen pipettiert. Anschließend werden 10 μl der Glyphosat‑2‑^13^C,^15^N‑Dotierlösung sowie 800 μl hochreines Wasser zugegeben. Die Proben werden in das mit Extrak­tionskartuschen (Strata SAX, 100 mg/ml) und Polypropylenröhrchen versehene ASPEC^®^‑System gestellt und wie unten beschrieben extrahiert.

**Table:** 

Konditionierung:	1 ml Methanol
	1 ml hochreines Wasser
Probe:	1 ml
Waschschritt:	1 ml hochreines Wasser
	1 ml Methanol
Trockenschritt:	lufttrocknen, bis die Probe nicht mehr tropft
Elutionsschritt:	1 ml 10%ige Ameisensäure in Methanol

Das Eluat wird im Heizblock bei 40 °C unter leichtem Stickstoffstrom zur Trockene eingeengt und der Rückstand in 100 μl 0,1%iger Ameisensäure wiederaufgenommen, gut gemischt und in das Kunststoff-Insert eines LC‑Gläschens überführt. Ein Aliquot von 10 μl wird für die Analyse verwendet.

#### Methodenprüfung

Vor der Analyse werden die Urinproben bei Raumtemperatur aufgetaut und gründlich gemischt. Von jeder Urin­probe wird 1 ml in ein Polypropylenröhrchen pipettiert. Anschließend werden 10 μl der Glyphosat‑2‑^13^C,^15^N- und AMPA‑^13^C,^15^N,D_2_‑Dotierlösung hinzugefügt. Die Proben werden dann nicht-automatisiert mittels SPE aufgereinigt. Dazu werden die SPE-Kartuschen mit 2 ml Methanol, gefolgt von 2 ml hochreinem Wasser, konditioniert. Nach Probenaufgabe werden die Kartuschen mit 2 ml hochreinem Wasser, gefolgt von 2 ml Methanol und 1 ml 10%iger Ameisensäure in Methanol, gewaschen. Die Analyten werden mit 1,5 ml 10%iger Ameisensäure in Methanol in Poly­propylenröhrchen eluiert.

Das Eluat wird im Heizblock bei 45 °C unter leichtem Stickstoffstrom zur Trockene eingeengt und der Rückstand in 100 μl 0,1%iger Ameisensäure in Wasser aufgenommen. Die rekonstituierte Lösung wird gründlich gemischt und in das Kunststoff-Insert eines LC-Gläschens überführt. Ein Aliquot von 10 μl wird für die Analyse verwendet.

## 6 Instrumentelle Arbeitsbedingungen

Während der Methodenentwicklung wurden die Analysen mit einem LC‑MS/MS-System durchgeführt, das aus einem Shimadzu HPLC-System und einem AB SCIEX 3200 Tandem-Massenspektrometer bestand. Die Prüfer der Methode verwendeten eine Agilent 1260 HPLC-Anlage und ein AB SCIEX 5500 QTRAP^®^ Tandem-Massenspektrometer.

Die in diesem Abschnitt für die beiden Gerätekonfigurationen beschriebenen Einstellungen sind gerätespezifisch und müssen vom Anwender getestet und gegebenenfalls angepasst werden. Die hier gegebenen Informationen sind daher nur als Anhaltspunkte zu verstehen. Bei Geräten anderer Hersteller können weitere Anpassungen erforderlich sein.

### 6.1 Hochleistungsflüssigkeitschromatographie

#### Methodenentwicklung

**Table:** 

Analytische Säule:	ZORBAX Eclipse XDB‑C8, 4,6 × 150 mm, 5 μm
Trennprinzip:	*Reversed phase*
Eluent:	A: 0,1 % Ameisensäure in Wasser
	B: Acetonitril
Injektionsvolumen:	10 μl
Flussrate:	0,4 ml/min
Gradientenprogramm:	siehe Tabelle 4

**Tab. 4:** Gradientenprogramm für die Bestimmung von Glyphosat in Urin (ZORBAX Eclipse XDB‑C8)

Zeit [min]	Eluent A [%]	Eluent B [%]
0	95	5
2	95	5
8	5	95
10	5	95
10,5	95	5
16	95	5

Ein Sechs-Wege-Ventil leitet den Fluss in den Abfall und nach zwei Minuten zur Ionenquelle.

#### Methodenprüfung I

**Table:** 

Analytische Säule:	ZORBAX RR Eclipse XDB‑C8, 2,1 × 150 mm, 3,5 μm
Trennprinzip:	*Reversed phase*
Säulentemperatur:	40 °C
Eluent:	A: 0,1 % Ameisensäure in Wasser
	B: Acetonitril
Injektionsvolumen:	10 μl
Flussrate:	0,2 ml/min
Gradientenprogramm:	siehe Tabelle 5

**Tab. 5:** Gradientenprogramm für die Bestimmung von Glyphosat und AMPA in Urin (ZORBAX RR Eclipse XDB‑C8)

Zeit [min]	Eluent A [%]	Eluent B [%]
0	100	0
2	100	0
8	5	95
10	5	95
11	100	0
20	100	0

#### Methodenprüfung II

**Table:** 

Analytische Säule:	Raptor Polar X, 50 × 2,1 mm, 2,7 μm
Trennprinzip:	Hydrophile Interaktionsflüssigkeitschromatographie (*hydrophilic interaction liquid chromatography,* HILIC) und Ionenaustausch
Säulentemperatur:	40 °C
Eluent:	A: 0,5 % Ameisensäure in Wasser
	B: 0,5 % Ameisensäure in Acetonitril
Injektionsvolumen:	10 μl
Flussrate:	0,5 ml/min
Gradientenprogramm:	siehe Tabelle 6

**Tab. 6:** Gradientenprogramm für die Bestimmung von Glyphosat und AMPA in Urin (Raptor Polar X)

Zeit [min]	Eluent A [%]	Eluent B [%]
0	35	65
1	35	65
4	90	10
9	90	10
10	35	65
15	35	65

### 6.2 Tandem-Massenspektrometrie

Auch die gerätespezifischen Parameter des Massenspektrometers müssen vom Anwender individuell für das eingesetzte MS/MS‑System ermittelt und eingestellt werden. Die in diesem Abschnitt genannten gerätespezifischen Parameter sind für die während der Methodenentwicklung und Methodenprüfung verwendeten Systeme bestimmt und optimiert worden.

#### Methodenentwicklung

**Table:** 

Detektionsmodus:	*Multiple Reaction Monitoring *(MRM)
Ionisierung:	Elektrospray, negativ (ESI−)
Quellentemperatur:	500 °C
Ionenspray-Spannung:	−4500 V
Gasfluss Curtain-Gas:	50 l/min
Ionenquelle, Gasfluss Gas 1:	70 l/min
Ionenquelle, Gasfluss Gas 2:	50 l/min
CAD (charged aerosol detection):	High
Dwelltime:	0,2 s
Parameterspezifische Einstellungen:	siehe Tabelle 7

**Tab. 7:** Parameterspezifische Einstellungen für die Bestimmung von Glyphosat in Urin (Methodenentwicklung)

Substanz	Retentionszeit ZORBAX Eclipse XDB-C8 [min]	Vorläufer-Ion (*m/z*)	Produkt-Ion (*m/z*)
Glyphosat	3,5	168	63
Glyphosat-2‑^13^C,^15^N (ISTD)	3,5	170	63

#### Methodenprüfung

**Table:** 

Detektionsmodus:	*Multiple Reaction Monitoring* (MRM)
Ionisierung:	Elektrospray, negativ (ESI−)
Quellentemperatur:	600 °C
Ionenspray-Spannung:	−4500 V
Gasdruck Curtain-Gas:	20 psi (1,38 bar)
Gasdruck Gas 1:	80 psi (5,52 bar)
Gasdruck Gas 2:	60 psi (4,14 bar)
CAD (charged aerosol detection):	Low
Dwelltime:	0,15 s
Eintrittspotenzial:	−10 V
Kollisionsenergie:	−20 V
Kollisionszellen-Austrittspotenzial:	−14 V
Parameterspezifische Einstellungen:	siehe Tabelle 8

**Tab. 8:** Parameterspezifische Einstellungen für die Bestimmung von Glyphosat und AMPA in Urin (Methodenprüfung)

Substanz	Retentionszeit ZORBAX RR Eclipse XDB-C8 [min]	Retentionszeit Raptor Polar X [min]	Vorläufer-Ion (*m/z*)	Produkt-Ion (*m/z*)
Glyphosat	1,87	10,87	168	63^a)^
		168	79^b)^
Glyphosat-2‑^13^C,^15^N (ISTD)	1,87	10,87	170	63
AMPA	2,02	1,44	110	63^a)^
		110	79^b)^
AMPA-^13^C,^15^N,D_2 _(ISTD)	2,02	1,44	114	63

^a)^ Quantifier

^b)^ Qualifier

## 7 Analytische Bestimmung

Es werden jeweils 10 μl der gemäß Abschnitt 5.2 aufgearbeiteten Probe in das LC‑MS/MS injiziert. Die analytische Trennung erfolgt mittels *Reversed‑Phase*-Chromatographie (ZORBAX Eclipse XDB‑C8‑Säule oder ZORBAX RR Eclipse XDB‑C8‑Säule) oder durch HILIC und Ionenaustausch (Raptor Polar X-Säule). Die Identifizierung der Analyten erfolgt auf der Grundlage der jeweiligen Retentionszeiten und der spezifischen Massenübergänge. Die in Tabelle 7 und 8 für die Analyten und ISTDs aufgeführten Retentionszeiten können nur als Anhaltspunkt dienen. Der Anwender hat sich selbst von der Trennleistung der verwendeten HPLC‑Säule und dem daraus resultierenden Retentionsverhalten der Analyten zu überzeugen.

**Abb. 2:**
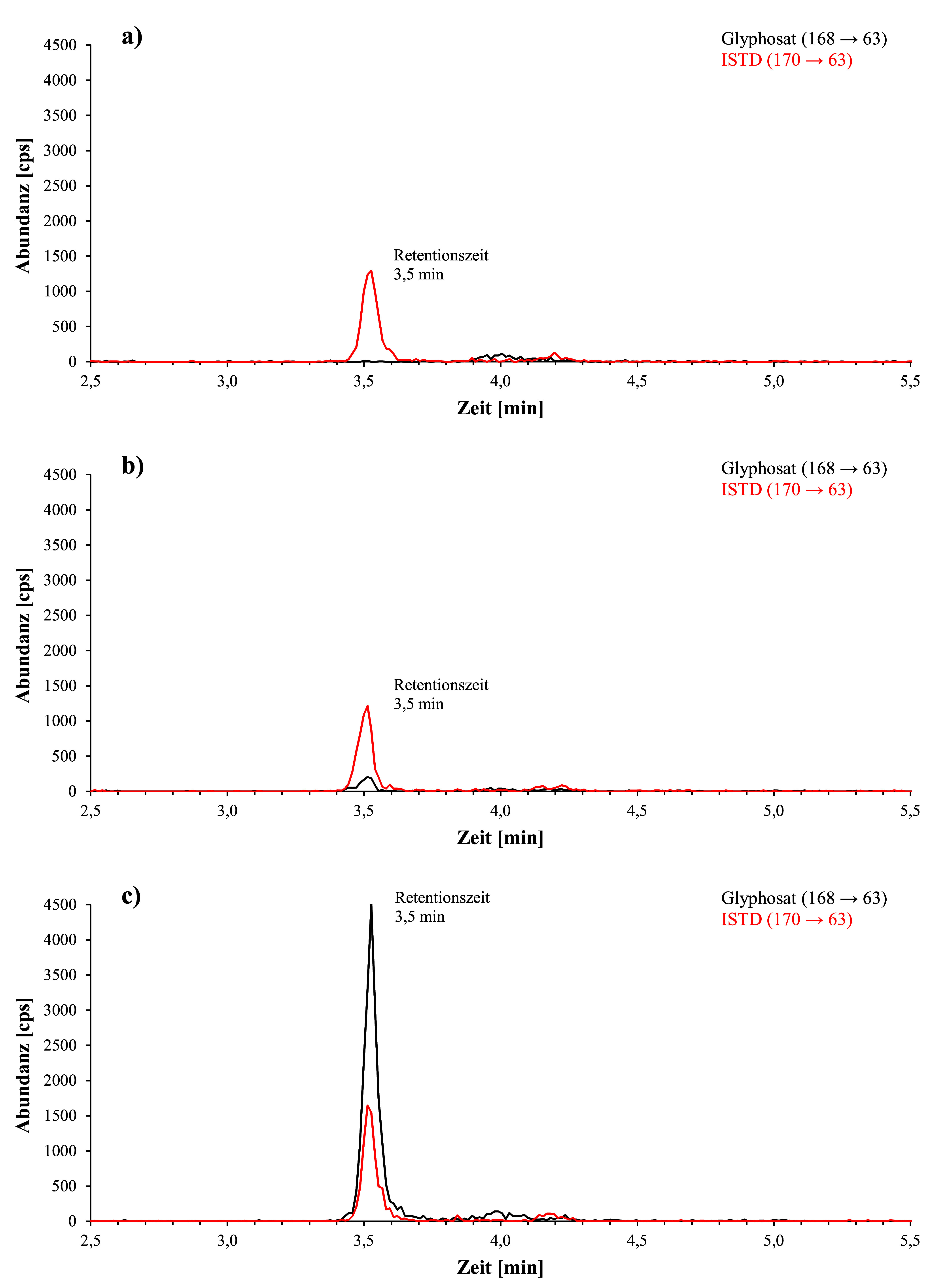
Bei der Methodenentwicklung erhaltene Chromatogramme a) einer Urinprobe einer Person ohne bekannte Glyphosatexposition (Glyphosatkonzentration < Nachweisgrenze), b) einer Urinprobe mit einem Hintergrundgehalt von 1,2 μg Glyphosat/l und c) eines Glyphosat-Kalibrierstandards (20 μg/l Urin)

**Abb. 3:**
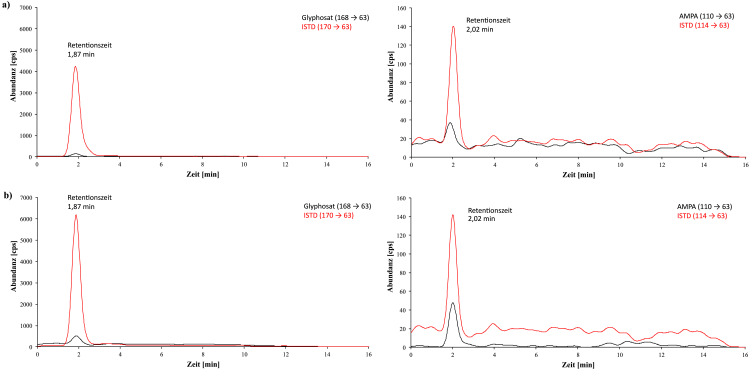
Bei der Methodenprüfung mit der ZORBAX RR Eclipse XDB‑C8-Säule erhaltene Chromatogramme a) einer Realprobe mit 1,4 μg Glyphosat/l Urin und 0,62 μg AMPA/l Urin, b) eines Kalibrierstandards mit 1 μg Glyphosat/l Urin und 1 μg AMPA/l Urin

**Abb. 4:**
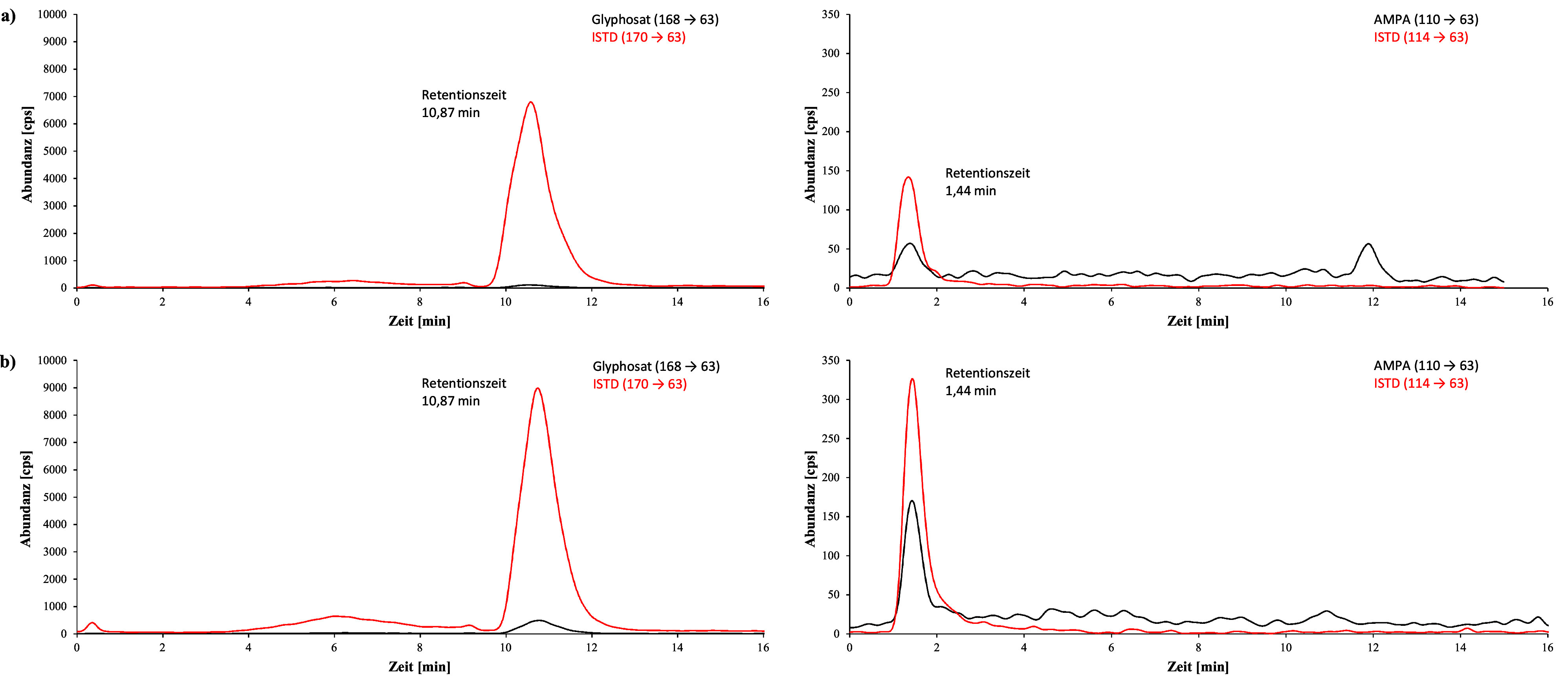
Bei der Methodenprüfung mit der Raptor Polar X‑Säule erhaltene Chromatogramme a) einer Realprobe mit 0,64 μg Glyphosat/l Urin und 0,62 μg AMPA/l Urin, b) eines Kalibrierstandards mit 1 μg Glyphosat/l Urin und 1 μg AMPA/l Urin

Abbildung 2 zeigt beispielhaft während der Methodenentwicklung aufgenommene Chromatogramme einer Urinprobe von einer Person ohne bekannte Glyphosatexposition, einer Urinprobe mit einem Hintergrundgehalt von 1,2 μg Glyphosat/l, sowie eines mit 20 μg Glyphosat/l Urin dotierten Kalibrierstandards. Die bei der Methodenprüfung mit der ZORBAX RR Eclipse XDB‑C8-Säule erhaltenen Chromatogramme einer nativen Urinprobe mit Hintergrundgehalten von 1,4 μg Glyphosat/l und 0,62 μg AMPA/l sowie eines mit 1 μg Glyphosat und 1 μg AMPA pro Liter Urin dotierten Kalibrierstandards sind in Abbildung 3 dargestellt. Abbildung 4 zeigt beispielhafte, bei der Methodenprüfung mit der Raptor Polar X‑Säule erhaltene Chromatogramme eines nativen Urins mit einem ermittelten Gehalt von 0,64 μg Glyphopsat/l und 0,62 μg AMPA/l sowie eines mit 1 μg Glyphosat und 1 μg AMPA pro Liter dotierten Kalibrierstandards.

## 8 Kalibrierung

**Abb. 5:**
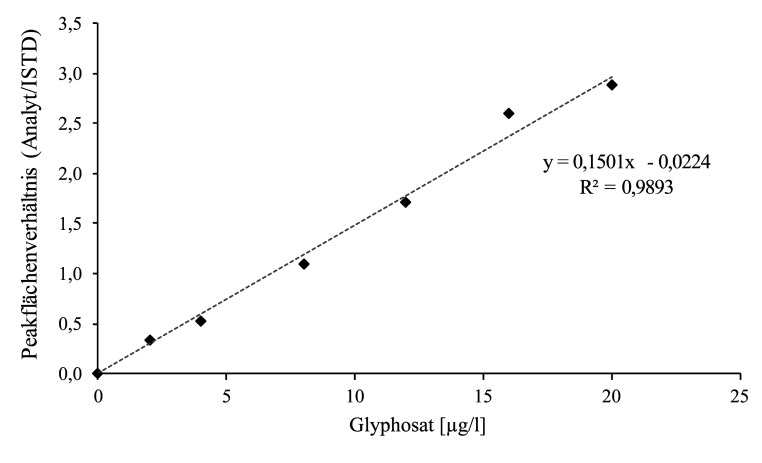
Beispielhafte Kalibriergerade für die Bestimmung von Glyphosat in Urin (Methodenentwicklung)

**Abb. 6:**
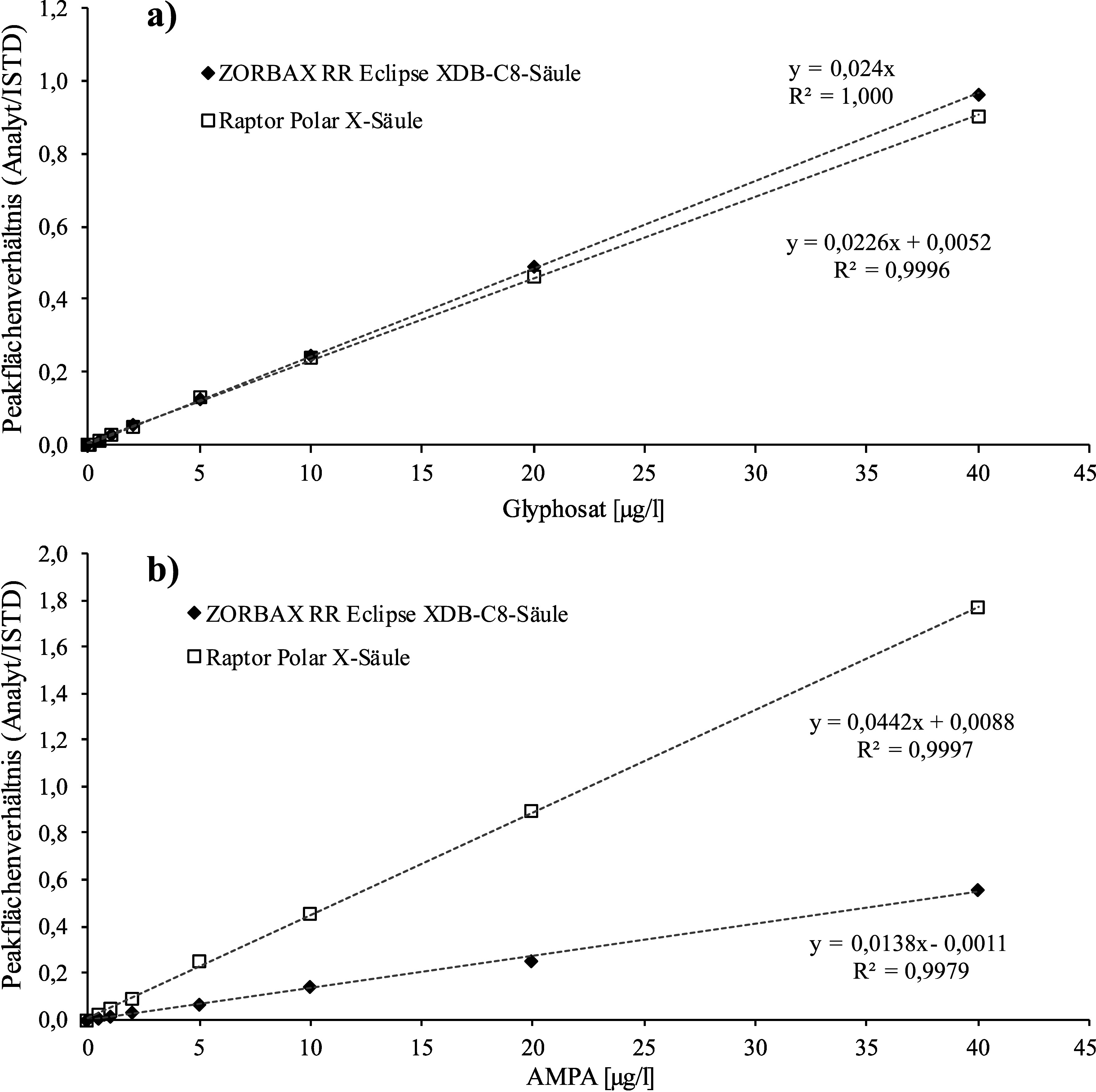
Beispielhafte Kalibriergeraden für die Bestimmung von Glyphosat und AMPA in Urin jeweils mit der a) ZORBAX RR Eclipse XDB-C8-Säule und der b) Raptor Polar X-Säule (Methodenprüfung)

Die in Abschnitt 4.5 beschriebenen Kalibrierstandards werden in der gleichen Weise wie die Proben hergestellt und aufgearbeitet (vgl. Abschnitt 5.2) und mittels LC‑MS/MS analysiert (vgl. Abschnitt 6). Die Kalibriergeraden werden durch Auftragen der Peakflächenverhältnisse von Analyt und zugehörigem ISTD gegen die jeweils dotierte Konzentration des Kalibrierstandards erstellt. Die Kalibriergerade ist im Konzentrationsbereich von der Nachweisgrenze bis 20 μg Glyphosat/l (Methodenentwicklung) oder bis 40 μg Glyphosat/l bzw. AMPA/l (Methodenprüfung) linear. Abbildung 5 zeigt beispielhaft eine von den Entwicklern der Methode erstellte Kalibriergerade für die Bestimmung von Glyphosat in Urin. Abbildung 6 zeigt exemplarische Kalibriergeraden für die Bestimmung von Glyphosat und AMPA im Urin, wie sie bei der Methodenprüfung unter Verwendung der ZORBAX RR Eclipse XDB-C8-Säule sowie mit der Raptor Polar X-Säule erstellt wurden.

## 9 Berechnung der Analysenergebnisse

Die Peakfläche des Analyten wird durch die Peakfläche des dazugehörigen ISTDs geteilt. Der so ermittelte Quotient wird in die Kalibrierfunktion (vgl. Abschnitt 8) eingesetzt und ergibt die jeweilige Analytkonzentration in μg/l. Da auch der Urin nicht exponierter Personen geringe Mengen Glyphosat und AMPA (hauptsächlich aus der Nahrung) enthält, werden die Peakflächen der Analyten nicht leerwertkorrigiert und die Steigung der Kalibriergerade wird zur Berechnung der Konzentration unbekannter Proben verwendet.

## 10 Standardisierung der Messergebnisse und Qualitätssicherung

Zur Qualitätssicherung der Analysenergebnisse wird gemäß den Richtlinien der Bundesärztekammer und den Angaben in dem von der Kommission veröffentlichten allgemeinen Kapitel verfahren (Bader et al. 2010; Bundesärztekammer 2014).

Materialien für die Qualitätskontrolle sind kommerziell nicht erhältlich, daher wird eine gepoolte Urinprobe mit einer bekannten Menge Glyphosat und AMPA dotiert. Die Qualitätskontrollproben werden – neben Reagenzienleerwerten und Urinleerwerten – in jedem Analyselauf gemessen. Die Qualität der Glyphosat- und AMPA-Bestimmung kann auch durch die Teilnahme am externen Qualitätssicherungsprogramm G‑EQUAS (*German External Quality Assessment Scheme*, https://www.g-equas.de/) gesichert werden.

## 11 Beurteilung des Verfahrens

Die ursprünglich entwickelte Methode ermöglichte die Quantifizierung von Glyphosat in Urin, der Glyphosatmetabolit AMPA war nicht in der Methode enthalten. Die Zuverlässigkeit dieses Verfahrens wurde durch eine umfassende Validierung sowie durch Nachstellung und Prüfung der Methode in einem zweiten, unabhängigen Labor bestätigt.

Neben der Nachstellung und Validierung der ursprünglichen Methode testeten die Prüfer eine alternative Säule und erweiterten die Methode um den Glyphosatmetaboliten AMPA. In den folgenden Abschnitten sind sowohl die Validierungsdaten der ursprünglich für Glyphosat entwickelten Methode als auch die von den Prüfern erhobenen Daten für Glyphosat und AMPA aufgeführt.

### 11.1 Präzision

#### Methodenentwicklung

Zur Bestimmung der Präzision in der Serie wurde Poolurin mit 2 μg und 7 μg Glyphosat pro Liter Urin dotiert, wie in Abschnitt 5.2 beschrieben parallel aufgearbeitet und analysiert (Abschnitt 6). Die von den Entwicklern der Methode erhaltenen Daten zur Präzision in der Serie sind in Tabelle 9 dargestellt.

**Tab. 9:** Präzision in der Serie für die Bestimmung von Glyphosat in Urin (n = 8 (2 μg/l), n = 10 (7 μg/l)); Methodenentwicklung

Analyt	Dotierte Konzentration [μg/l]	Analytische Säule	Standardabweichung (rel.) *s_w_* [%]	Streubereich *u* [%]
Glyphosat	2	ZORBAX Eclipse XDB‑C8	6,5	15,4
	7		2,7	6,11

Um die Präzision von Tag zu Tag zu bestimmen, wurde mit 4 μg Glyphosat pro Liter dotierter Urin über vier Jahre hinweg vielfach aufgearbeitet und analysiert. Die von den Entwicklern erzielten Präzisionsdaten sind in Tabelle 10 aufgeführt.

**Tab. 10:** Präzision von Tag zu Tag für die Bestimmung von Glyphosat in Urin (n = 381); Methodenentwicklung

Analyt	Dotierte Konzentration [μg/l]	Analytische Säule	Standardabweichung (rel.) *s_w_* [%]	Streubereich *u* [%]
Glyphosat	4	ZORBAX Eclipse XDB‑C8	9,8	19,4

#### Methodenprüfung

Bei der Methodenprüfung wurden die Daten zur Präzision in der Serie sowie zur Präzision von Tag zu Tag auf einer ZORBAX RR Eclipse XDB‑C8-Säule sowie einer Raptor Polar X-Säule erhoben. Die verwendeten Urine waren mit 2,5 μg bzw. 25 μg Glyphosat und AMPA/l dotiert. Die so erhaltenen Daten sind in Tabelle 11 und 12 gezeigt.

**Tab. 11:** Präzision in der Serie für die Bestimmung von Glyphosat und AMPA in Urin (n = 5); Methodenprüfung

Analyt	Dotierte Konzentration [μg/l]	Analytische Säule	Standardabweichung (rel.) *s_w_* [%]	Streubereich *u* [%]
Glyphosat	2,5	ZORBAX RR Eclipse XDB‑C8	2,4	6,7
	25		2,2	6,1
AMPA	2,5		3,5	9,7
	25		1,9	5,3
Glyphosat	2,5	Raptor Polar X	2,1	5,8
	25		1,3	3,6
AMPA	2,5		2,8	7,8
	25		3,8	10,5

**Tab. 12:** Präzision von Tag zu Tag für die Bestimmung von Glyphosat und AMPA in Urin (n = 3); Methodenprüfung

Analyt	Dotierte Konzentration [μg/l]	Analytische Säule	Standardabweichung (rel.) *s_w_* [%]	Streubereich *u* [%]
Glyphosat	2,5	ZORBAX RR Eclipse XDB‑C8	3,4	14,6
	25		2,4	10,3
AMPA	2,5		5,4	23,2
	25		2,6	11,2
Glyphosat	2,5	Raptor Polar X	4,3	18,5
	25		2,1	9,0
AMPA	2,5		3,1	13,3
	25		4,2	18,1

### 11.2 Richtigkeit

#### Methodenentwicklung

Um den Einfluss der Urinmatrix zu ermitteln, wurden von den Entwicklern der Methode fünf Individualurine sowohl undotiert als auch mit 4 μg bzw. 20 μg Glyphosat pro Liter dotiert gemessen. Die Ergebnisse für die mittlere relative Wiederfindung sind in Tabelle 13 dargestellt.

**Tab. 13:** Mittlere relative Wiederfindung für die Bestimmung von Glyphosat in Individualurinen (n = 5); Methodenentwicklung

Analyt	Dotierte Konzentration [μg/l]	Analytische Säule	Mittlere Wiederfindung (rel.) *r* [%]	Bereich [%]
Glyphosat	4	ZORBAX Eclipse XDB‑C8	94	91–100
	20		98	95–102

#### Methodenprüfung

Bei der Prüfung der Methode wurde die Richtigkeit der Bestimmung von Glyphosat und AMPA in Urin aus den Daten der Präzision von Tag zu Tag errechnet. Die Ergebnisse für die mittlere relative Wiederfindung sind in Tabelle 14 dargestellt.

**Tab. 14:** Mittlere relative Wiederfindung für die Bestimmung von Glyphosat und AMPA in Urin (n = 3); Methodenprüfung

Analyt	Dotierte Konzentration [μg/l]	Analytische Säule	Mittlere Wiederfindung (rel.) *r* [%]	Bereich [%]
Glyphosat	2,5	ZORBAX RR Eclipse XDB‑C8	99	96–103
	25		100	98–101
AMPA	2,5		102	97–107
	25		102	101–104
Glyphosat	2,5	Raptor Polar X	102	96–106
	25		100	99–102
AMPA	2,5		106	104–107
	25		100	97–105

### 11.3 Nachweis- und Bestimmungsgrenzen

Im Rahmen der Methodenentwicklung wurde die Nachweisgrenze aus dem dreifachen Signal/Rausch-Verhältnis und die Bestimmungsgrenze aus dem zehnfachen Signal/Rausch-Verhältnis errechnet. Die Prüfer der Methode berechneten die Bestimmungsgrenze als Blindwert plus fünffache Standardabweichung des Blindwertes. Die berechneten Werte sind in Tabelle 15 (Methodenentwicklung) und Tabelle 16 (Methodenprüfung) aufgeführt.

#### Methodenentwicklung

**Tab. 15:** Nachweis- und Bestimmungsgrenze für die Bestimmung von Glyphosat in Urin; Methodenentwicklung

Analyt	Analytische Säule	Nachweisgrenze [μg/l]	Bestimmungsgrenze [μg/l]
Glyphosat	ZORBAX Eclipse XDB‑C8	0,15	0,5

#### Methodenprüfung

**Tab. 16:** Nachweis- und Bestimmungsgrenzen für die Bestimmung von Glyphosat und AMPA in Urin; Methodenprüfung

Analyt	Analytische Säule	Nachweisgrenze [μg/l]	Bestimmungsgrenze [μg/l]
Glyphosat	ZORBAX RR Eclipse XDB‑C8	0,1	0,5
AMPA		0,1	0,5
Glyphosat	Raptor Polar X	0,05	0,1
AMPA		0,1	0,5

### 11.4 Störeinflüsse

Die Bestimmung von Glyphosat und AMPA in Humanurin ist anspruchsvoll und stellt hohe Anforderungen an die Robustheit des LC‑MS/MS-Systems. Eine gründliche Reinigung zwischen den Analysenserien ist sehr zu empfehlen, insbesondere, wenn umfangreiche Probenserien gemessen wurden.

Um bei der Bestimmung von Glyphosat und AMPA in Urin möglichst niedrige Bestimmungsgrenzen zu erreichen, ist die Wahl der Trennsäule von entscheidender Bedeutung. Die mit den RP-Säulen (ZORBAX Eclipse XDB‑C8 und ZORBAX RR Eclipse XDB‑C8) erreichten Bestimmungsgrenzen von 0,5 μg/l sind ausreichend, um eine berufliche Exposition und hohe Hintergrundgehalte in der Allgemeinbevölkerung zu erfassen. Mit der von den Prüfern der Methode getesteten Raptor Polar X-Säule, deren Trennprinzip auf der Kombination von HILIC und Ionenaustausch beruht, fand sich aber für Glyphosat eine um den Faktor fünf niedrigere Bestimmungsgrenze. Dies lag vor allem an der auf dieser Säule deutlich längeren Retentionszeit des Glyphosats. Dadurch wurden die auf den RP-Säulen bei Retentionszeiten unter dreieinhalb Minuten auftretenden Matrixeinflüsse durch nicht zurückgehaltene Salze und kleine Moleküle vermindert.

Bekannt ist, dass Glyphosat und AMPA leicht von Glasoberflächen adsorbiert werden können (Alferness et al. 2001). Um die Adsorption von Glyphosat und AMPA an Glasoberflächen zu vermeiden, sollten die Urinproben und Standardlösungen stets in Plastikbechern oder -röhrchen aufbewahrt werden. Zudem sollten die Analyten beim Ansetzen von Lösungen und Kalibrierstandards auf vorgelegtes Lösemittel pipettiert werden. Allerdings weisen Hoppe et al. (2023) auch auf mögliche Störungen der Glyphosat- und AMPA-Analytik durch in Polypropylenröhrchen enthaltene Kunststoffadditive hin.

Bei dem Aufreinigungs- und Anreicherungsschritt mittels Festphasenextraktion wurde von den Prüfern der Methode für den letzten Waschschritt sowie für die Elution der Analyten 10%ige Ameisensäure verwendet. Die Prüfer der Methode haben die jeweils eluierenden Fraktionen der einzelnen SPE-Schritte analysiert und nachgewiesen, dass der Waschschritt unter Verwendung der 10%igen Ameisensäure zu keinen Analytverlusten führt, während mit dem Elutionsschritt die Analyten vollständig von der Säule gespült werden. Das Einengen der Probenlösungen im Anschluss an die SPE ist unkritisch, da die sehr polaren Analyten nicht flüchtig sind.

## 12 Diskussion der Methode

Die vorliegende Analysenmethode wurde entwickelt, um Glyphosat im Urin von Beschäftigten zu quantifizieren (Connolly et al. 2018). Dabei erlaubt die von den Entwicklern der Methode verwendete automatisierte SPE einen hohen Probendurchsatz und ist somit für den Routinebetrieb und große Probenserien sehr gut geeignet. Mit einer Bestimmungsgrenze von 0,5 μg Glyphosat pro Liter Urin können berufliche Expositionen zuverlässig erfasst werden (Connolly et al. 2019). Die Bestimmungsgrenze für Glyphosat ist vergleichbar mit den von anderen Arbeitsgruppen für LC-Methoden publizierten Werten: so wurden ohne Derivatisierung Bestimmungsgrenzen von 0,1 bis 0,5 μg/l (Li und Kannan 2022; Nomura et al. 2020; Ruiz et al. 2021) und mit Derivatisierung Bestimmungsgrenzen von 0,25 bis 1 μg/l ermittelt (Bienvenu et al. 2021; Bressán et al. 2021; Martin-Reina et al. 2021).

Bei der Methodenprüfung wurde zusätzlich zum Glyphosat auch dessen einziger Metabolit AMPA in die Methode integriert. Da die Verwendung derselben sowie einer ähnlichen Säule wie bei der Methodenentwicklung zu einer deutlich kürzeren Retentionszeit des Glyphosats am LC-MS/MS-System der Prüfer führte, wurden weitere Säulen getestet (Polledri et al. 2023) und die Methode auch unter Verwendung einer alternativen Säule validiert. Diese alternative Säule, deren Trennprinzip auf der Kombination von HILIC und Ionenaustausch beruht, führte zu einer besseren Trennung der Analyten und zu einer deutlich verlängerten Retentionszeit des Glyphosats (siehe Tabelle 8).

Die Zuverlässigkeitskriterien der Methode sind für Glyphosat und auch für AMPA als sehr gut zu bezeichnen. Die Richtigkeit wurde durch die gute Wiederfindung nach Dotierung von gepooltem Urin als auch von Individualurinen belegt.

Zudem wurde für Glyphosat die Richtigkeit der Analysenergebnisse durch den Vergleich mit einer GC-Methode belegt. Hierzu wurden mit der vorliegenden LC‑MS/MS‑Methode (Health and Safety Executive, Harpur Hill, Buxton, Vereinigtes Königreich; Methodenentwicklung) und einer GC‑MS/MS‑Methode (Hoppe et al. 2023) 33 native Urinproben im Rahmen eines Interlaborvergleichs analysiert. Die Glyphosatkonzentrationen lagen bei 20 Proben oberhalb der Bestimmungsgrenzen beider Methoden. Die Messergebnisse dieser Proben wiesen eine sehr gute Korrelation auf (siehe Hoppe et al. 2023). AMPA wurde in dem Interlaborvergleich nicht bestimmt.

Die Empfindlichkeit der beschriebenen Methode reicht aus, um eine berufsbedingte Exposition gegen Glyphosat zu erfassen. Unter Verwendung der Raptor Polar X‑Säule reichen die ermittelten Bestimmungsgrenzen von 0,1 μg Glyphosat/l Urin und 0,5 μg AMPA/l Urin auch aus, höhere nicht-berufsbedingte Belastungen mit Glyphosat, z. B. durch die Ernährung, zu erfassen.

**Verwendete Messgeräte bei der Methodenentwicklung **LC‑MS/MS-System: Shimadzu LC‑20AB (Shimadzu UK Limited, Milton Keynes, Vereinigtes Königreich) mit einem AB SCIEX API 3200 Tandem-Massenspektrometer (AB SCIEX LLC, Framingham, MA, USA); C18-Vorsäule (Phenomenex Ltd, Macclesfield, Vereinigtes Königreich); analytische Säule: ZORBAX Eclipse XDB‑C8, 4,6 × 150 mm, 5 μm (Agilent Technologies LDA UK Limited, Stockport, Vereinigtes Königreich)

**Verwendete Messgeräte bei der Methodenprüfung **LC‑MS/MS-System: Agilent 1260 Infinity III (Agilent Technologies Italia S.p.A., Cernusco sul Naviglio, Italien) mit einem QTRAP^®^ 5500 (AB SCIEX LLC, Framingham, MA, USA); analytische Säulen: ZORBAX RR Eclipse XDB‑C8, 2,1 × 150 mm, 3,5 μm (Agilent Technologies Italia S.p.A., Cernusco sul Naviglio, Italien) und Raptor Polar X, 50 × 2,1 mm, 2,7 μm (Restek S.r.l., Cernusco sul Naviglio, Italien)
